# Electrochemical Lithiation and Delithiation of Amorphous Nonstoichiometric Silicon Oxide Thin-Film Electrode Studied by *Operando* X‑ray Photoelectron Spectroscopy

**DOI:** 10.1021/acs.jpclett.5c04065

**Published:** 2026-02-04

**Authors:** Tsukasa Iwama, Ryosuke Sugimoto, Raimu Endo, Tsuyoshi Ohnishi, Masakazu Haruta, Takayuki Doi, Takuya Masuda

**Affiliations:** † Research Center for Energy and Environmental Materials (GREEN), National Institute for Materials Science (NIMS), Tsukuba, Ibaraki 305-0044, Japan; ‡ Graduate School of Chemical Sciences and Engineering, Hokkaido University, Sapporo, Hokkaido 060-0810, Japan; § Department of Molecular Chemistry and Biochemistry, 68263 Doshisha University, Kyotanabe, Kyoto 610-0321, Japan; ∥ Department of Electric and Electronic Engineering, 26325 Kindai University, Iizuka, Fukuoka 820-8555, Japan

## Abstract

Electrochemical lithiation/delithiation of a nonstoichiometric silicon oxide (SiO_*x*_) thin-film electrode on a Li_6.6_La_3_Zr_1.6_Ta_0.4_O_12_ were analyzed using *operando* X-ray photoelectron spectroscopy (XPS). At the pristine SiO_*x*_ surface, bulk Si and SiO_*x*_ peaks were observed and remained unchanged in the capacity density from 0 to ∼1300 mAh g_Si_^–1^. At the capacity density of ∼1400 mAh g_Si_^–1^, however, new peaks corresponding to Li_∼2.0_Si and Li silicates appeared simultaneously with a substantial decrease in the bulk Si and SiO_*x*_ peaks. These results imply that, in the initial stage, lithiation of SiO_*x*_ occurred at the SiO_*x*_/Li_6.6_La_3_Zr_1.6_Ta_0.4_O_12_ interface to form Li_*y*_Si and Li silicates, which was beyond the probing depth of XPS. Subsequently, lithiation gradually propagated into the bulk and approached the probing depth of XPS as the composition reached Li_∼2.0_Si, thereby elongating the ion conductive pathway. Thereafter, the position of the Li_*y*_Si peak reversibly responded to the state of charge because lithiation/delithiation occurred uniformly across the SiO_*x*_ thin film.

## Graphical abstract

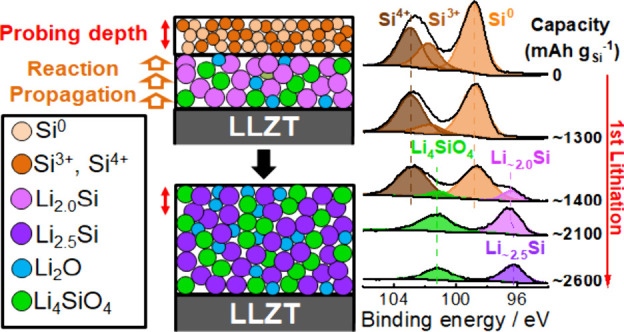

Pure Si anodes have an excellent theoretical capacity density of 4200 mAh g^–1^ corresponding to Li_4.4_Si (3579 mAh g^–1^ corresponding to Li_3.75_Si at room temperature).^1–6^ However, they undergo considerable (∼300%) volume change initiated by crystal structure changes during electrochemical lithiation/delithiation cycles, which causes mechanical failure of the composite electrode, leading to poor cycle performance.^7–11^ Nonstoichiometric silicon oxide (SiO_*x*_) shows very large irreversible capacity loss in the initial cycle because part of electrochemically inserted Li is consumed for the formation of electrochemically inactive species, such as Li_2_O and Li silicates.^12–15^ However, SiO_*x*_ anodes demonstrate significantly improved cycle performance as compared to that of pure Si anodes.^16–20^

To date, electrochemical lithiation/delithiation mechanisms of SiO_*x*_ electrodes have been explored by various research groups.^12–26^ Kim et al. used *ex-situ* solid-state nuclear magnetic resonance (NMR) to examine silicon monoxide (SiO) in a liquid-based electrolyte. They confirmed reversible lithiation and delithiation of Si domains, to form amorphous lithium silicide (*a*-Li_*y*_Si), as well as the irreversible lithiation of SiO_2_ domains to form highly resistive species such as Li silicates and Li_2_O.^14^ Takezawa et al. investigated the morphology and elemental composition of a SiO_*x*_ film electrode after repeated electrochemical cycles in a liquid-based electrolyte, using scanning electron microscopy (SEM) and electron probe microanalysis (EPMA). They proposed that the improved cycle performance of the SiO_*x*_ film electrode originates from the formation of Li silicates and Li_2_O which act as buffer layers against the volume change of Li_*y*_Si.^20^

In our previous study, electrochemical lithiation/delithiation reaction of an *a*-Si thin-film electrode sputter-deposited on a Li_6.6_La_3_Zr_1.6_Ta_0.4_O_12_ (LLZT) solid electrolyte in a Si/LLZT/Li configuration was analyzed by *operando* X-ray photoelectron spectroscopy (XPS).^27^ Although XPS measurements were performed at the *a*-Si surface opposite the Si/LLZT interface, upon lithiation, the Si 2p peak drastically shifted to a lower binding energy due to the formation of *a*-Li_*y*_Si, indicating a spatially uniform distribution of lithiation reaction across the entire electrode. Then, as the capacity increased, the Li_*y*_Si peak monotonically shifted to a lower binding energy. When the lithiation stopped at a capacity of 2200 mAh g^–1^, the Li_*y*_Si peak monotonically shifted to a higher binding energy throughout subsequent delithiation. When Li was inserted into the Li_*y*_Si up to a capacity of 3400 mAh g^–1^, where crystalline-Li_3.75_Si (*c*-Li_3.75_Si) and *a*-Li_*y*_Si coexisted, however, the Li_*y*_Si peak drastically shifted to a higher binding energy in the region of Li content *y* = 2.0–1.6 in the subsequent delithiation due to the phase transition from the *c*-Li_3.75_Si to *a*-Li_*y*_Si.^27^

In the present study, we investigated the mechanism of electrochemical lithiation/delithiation of a sputter-deposited *a*-SiO_*x*_ thin-film electrode on a LLZT solid electrolyte using *operando* XPS. Analysis based on the correlation between Li_*y*_Si peak positions and Li content *y*, as reported in our previous study,^27^ enabled the identification of Li_*y*_Si phases at each stage. This revealed that, in *a*-SiO_*x*_, the reaction initially occurs only in the vicinity of the SiO_*x*_/LLZT interface, and subsequently propagates throughout the entire thin film as ion conductive pathways of Li_∼2.0_Si are formed. Thereafter, electrochemical lithiation/delithiation reversibly occurs while maintaining a spatially uniform Li composition across the thin film. In addition, electrochemical lithiation of Li_*y*_Si was found to spontaneously cease at lower Li content as compared to pure Si electrode probably due to the formation of highly resistive byproducts such as Li_2_O and Li silicates.

An *a*-SiO_*x*_ thin film with a diameter of 10 mm and thickness of around 100 nm was deposited on a LLZT sheet (10 mm × 10 mm × 500 μm; Toshima Manufacturing Co., Ltd.) by radio frequency magnetron sputtering with Ar/O_2_ gas mixtures.^16,18^ Then, a Cu current collector was deposited onto the SiO_*x*_ layer by direct current sputtering, with a 10 mm × 4 mm central area masked by a stainless-steel stencil plate to leave an uncoated SiO_*x*_ region for XPS measurements. Finally, a Li metal layer with a thickness of around 1.5 μm was thermally evaporated onto the opposite surface of the LLZT sheet to yield a Cu/SiO_*x*_/LLZT/Li all-solid-state (ASS) half-cell, as shown in Figure 1.

**Figure 1:**
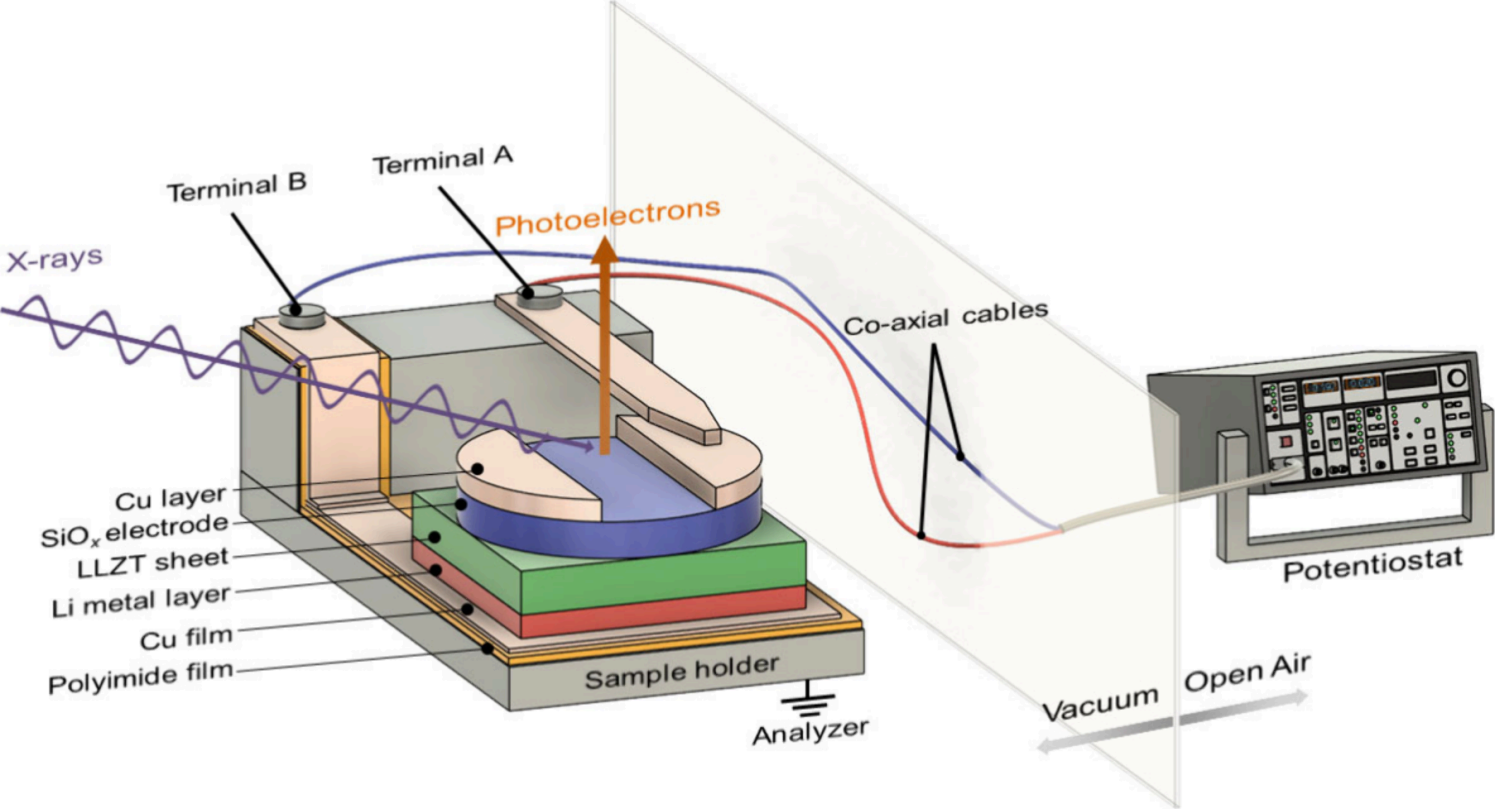
Schematic illustration of a Cu/SiO_*x*_/LLZT/Li ASS half-cell mounted onto a sample holder connected to a potentiostat for *operando* XPS measurements.

Electrochemical lithiation/delithiation and XPS measurements were performed simultaneously using an *operando* XPS system.^27–29^ Lithiation of the SiO_*x*_ thin-film electrode of the ASS half-cell was conducted in constant-current (CC)–constant-voltage (CV) mode: a constant current of 4.9 μA cm^–2^ (∼0.134 C, 1 C = 3579 mA g^–1^ for Li_3.75_Si) was applied until the cell voltage reached 0.02 V (109 min), followed by maintaining 0.02 V until the current density decreased to 0.49 μA cm^–2^ (546 min). Delithiation was carried out with a constant-current (CC) mode: a constant current of 2.45 μA cm^–2^ (∼0.067 C) was applied until the cell voltage reached 1.5 V (285 min). Subsequent lithiation/delithiation cycles followed the same procedure, but the current densities for CC and CV for lithiation were set to half of those for the first lithiation.

Figure 2 (a) shows the lithiation/delithiation potential profiles obtained for the ASS half-cell. The capacity densities are given in mAh g_Si_^–1^ based on the weight of Si in the *a*-SiO_*x*_ thin-film electrode. It is noted that the composition of *a*-SiO_*x*_ in the present study was determined to be *a*-SiO_0.5_ (see below). The first lithiation and delithiation capacity densities were 2625 and 1148 mAh g_Si_^–1^, respectively. The initial Coulombic efficiency of the SiO_*x*_ thin film, 43.7%, was much lower than those of previous reports on pure Si thin-film electrode in both liquid-electrolyte-based^3,30,31^ and all-solid-state configurations^27,29,32,33^ but in reasonable agreement with that of SiO_*x*_ in a liquid-based electrolyte.^23^ In the second cycle, the lithiation and delithiation capacity densities were 1276 and 1185 mAh g_Si_^–1^, respectively, with a Coulombic efficiency of 92.8%.

**Figure 2:**
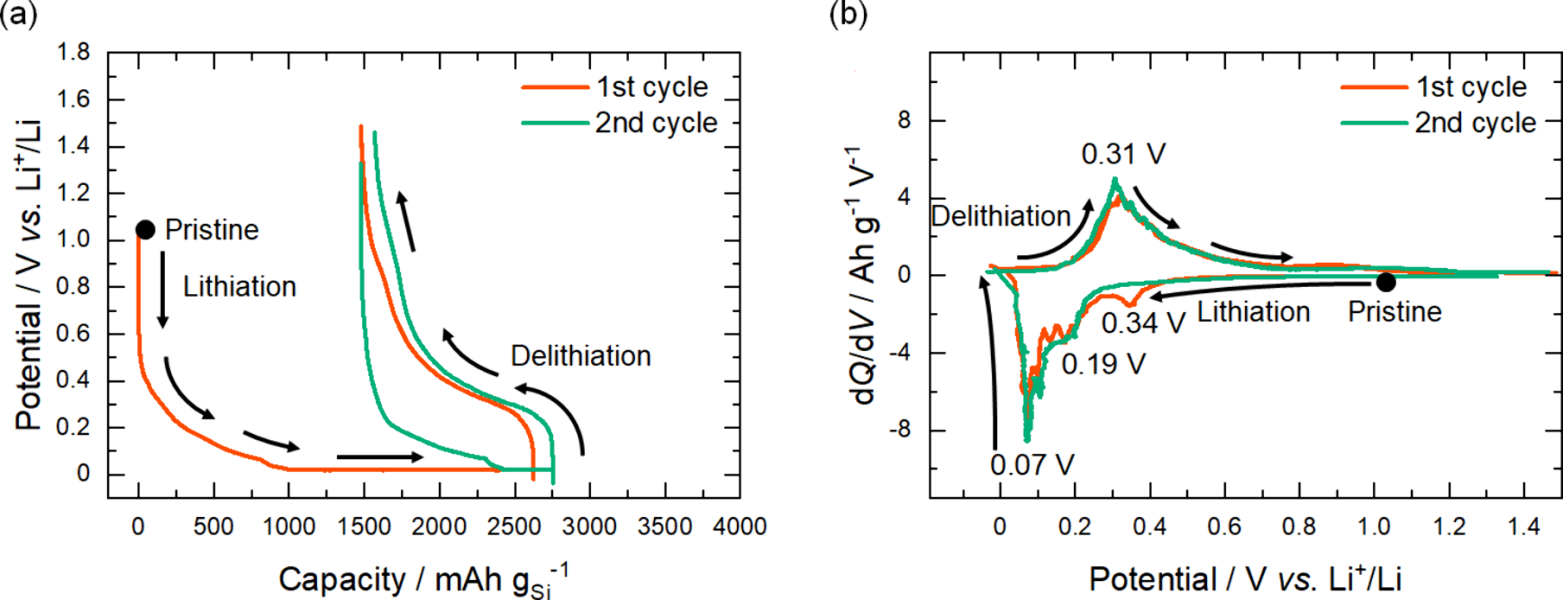
(a) Potential profiles and (b) generated d*Q*/d*V* curves during the first and second lithiation/delithiation processes of the *a*-SiO_*x*_ thin-film electrode in the Cu/SiO_*x*_/LLZT/Li ASS half-cell. Specific capacity was normalized by the weight of deposited Si in the *a*-SiO_*x*_ thin-film electrode.

Figure 2 (b) shows negative and positive d*Q*/d*V* curves obtained by differentiating the smoothed potential profiles during lithiation and delithiation, respectively. In the negative d*Q*/d*V* curve during the first lithiation, three peaks observed at around 0.34, 0.19, and 0.07 V were attributed to lithiation of *a*-SiO_*x*_ to form Li_2_O and Li silicates,^13,14,19^ lithiation of Si to form Li-poor *a*-Li_*y*_Si,^19^ and lithiation of the Li-poor *a*-Li_*y*_Si to form Li-rich *a*-Li_*y*_Si,^13,19,21^ respectively. The positive d*Q*/d*V* curve during the first delithiation showed a broad peak at around 0.31 V corresponding to delithiation of Li-rich *a*-Li_*y*_Si.^19^

In the second lithiation, the negative peak corresponding to lithiation of SiO_*x*_ observed at around 0.34 V in the first lithiation completely disappeared, confirming elimination of SiO_*x*_ to form Li_2_O and Li silicates in the first lithiation.^13,14,19^ Except for this point, the positive and negative d*Q*/d*V* curves remained almost unchanged thereafter, indicating reversible lithiation/delithiation processes.

Figure 3 shows the Li 1s and Si 2p photoelectron spectra of the *a*-SiO_*x*_ thin-film electrode after the first and second lithiation/delithiation processes. In the pristine state, Si 2p_3/2_ peaks observed at around 98.7 eV were attributed to the bulk Si (Si^0^), while a broad peak observed at around 99.7–102.7 eV was deconvoluted into two peaks of Si_2_O_3_ (Si^3+^) at 101.7 eV and SiO_2_ (Si^4+^) at 102.7 eV as a result of curve fitting analysis with the Voigt function, as shown in Figure S1.^17,23,34–36^ Although the curve fitting analysis was performed based on an assumption that the Si 2p spectra in SiO_*x*_ were composed of five species with the oxidation states Si^0^, Si_2_O (Si^1+^), SiO (Si^2+^), Si_2_O_3_ (Si^3+^), and SiO_2_ (Si^4+^), the best fit was obtained with three pairs of peaks consisting of Si^0^, Si^3+^ and Si^4+^ species. The details for the curve fitting analysis in the Si 2p region are described in the Supporting Information.

**Figure 3:**
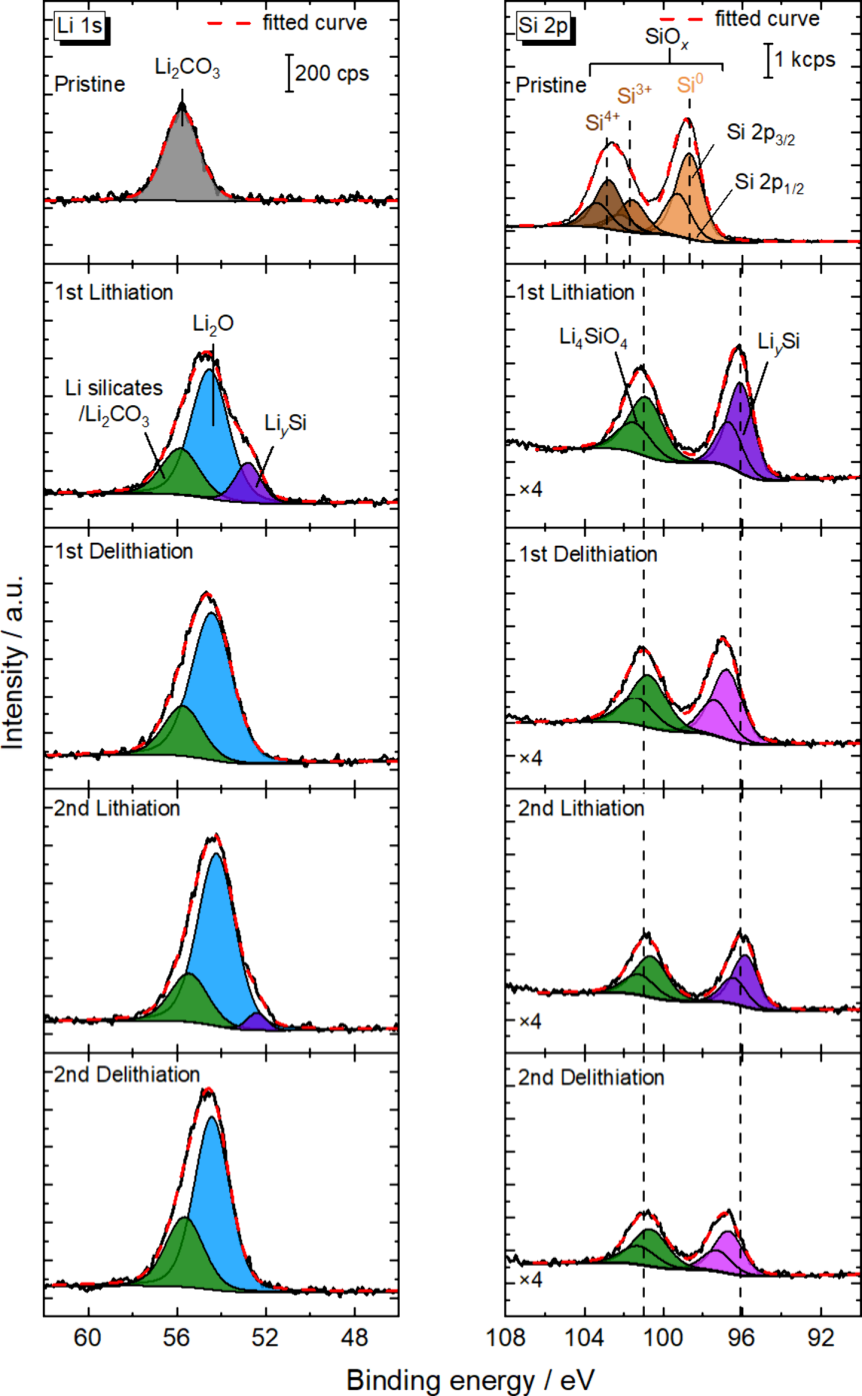
Li 1s and Si 2p photoelectron spectra of the *a*-SiO_*x*_ thin-film electrode in a Cu/SiO_*x*_/LLZT/Li ASS half-cell after the first and second lithiation and delithiation.

The composition of SiO_*x*_ in the pristine state was estimated to be SiO_0.87_, SiO_0.50_ and SiO_0.53_ by XPS using an Al Kα source, HAXPES using a Cr Kα source, and SEM-EDS analysis, respectively, as shown in Figure S1 and Table S1 in the Supporting Information. Even if the samples were handled only in a vacuum chamber or Ar-filled glovebox, the growth of Si oxides still occurs.^29^ In addition, such an outermost oxide layer becomes more dominant in photoelectron spectra measured using an Al Kα source due to its relatively high surface sensitivity. On the other hand, techniques with greater information depth, such as HAXPES and SEM-EDS, are considered to provide results that reflect the composition of the entire SiO_*x*_ layer. Thus, the value *x* in SiO_*x*_ used in this study is most likely ∼0.5.

It is noted that the Si^0^ peak was observed at a binding energy slightly lower than those of *a*-Si thin-film electrode in the pristine state (99.1 eV)^27^ and commercially available pure Si (99.2 eV).^37^ Based on the correlation between the position of Si 2p_3/2_ peak and Li content *y* in Li_*y*_Si in Table S2 and Figure S2 in the Supporting Information,^27^ the peak at 98.7 eV was attributed to Li_0–0.02_Si. In addition, a peak corresponding to Li_2_CO_3_ was observed in the Li 1s and C 1s regions in the pristine state.^38^ These results suggest that a small amount of Li species introduced as impurities during the sample preparation procedure can be inserted into Si^0^ of the SiO_*x*_ thin film to form slightly lithiated species, Li_0–0.02_Si. Subsequently, part of Li_0–0.02_Si further reacted with residual gases such as O_2_ and CO_2_ present in the vacuum chamber.

After the first lithiation, in addition to the Li_2_CO_3_ peak at 55.9 eV which cannot be deconvoluted from Li silicates, species assignable to Li_*y*_Si and Li_2_O were observed at around 52.8 and 54.5 eV in the Li 1s region, respectively. In the Si 2p region, a Si 2p_3/2_ peak corresponding to Li_0–0.02_Si shifted to a lower binding energy up to 96.1 eV which is equivalent to Li_2.53_Si based on the correlation in Figure S2,^27^ confirming the increase in Li content.^28,39^ In addition, the peaks corresponding to Si^3+^ species at around 101.7 eV and Si^4+^ species at around 102.7 eV merged into a peak at around 100.9 eV due to the formation of Li_4_SiO_4_.^23,29,39–41^ It is worth noting that the intensities of Si 2p peaks became much smaller than those of the pristine state because the atomic density of Si decreased due to the lithiation. Moreover, photoelectrons ejected from Li_*y*_Si were significantly attenuated by the topmost layer composed of Li_2_O, Li_2_CO_3_, and Li_4_SiO_4_. Based on an assumption that all the electrochemical charge was consumed for the lithiation of Si atoms contained in the *a*-SiO_*x*_ thin film, Li_2.75_Si should be formed during the first lithiation with the capacity density of 2625 mAh g_Si_^–1^, and thus around 8% of Li ions were consumed for the formation of Li_4_SiO_4_ and Li_2_O.

After the first delithiation, the Li_*y*_Si peak at around 52.8 eV disappeared from the Li 1s region. Moreover, the shift of the Li_*y*_Si peak from 96.1 to 97.0 eV in the Si 2p region confirms the decrease of Li content from Li_2.53_Si to Li_1.20_Si using the correlation in Figure S3.^27,39^ In contrast, the Li_2_O and Li_2_CO_3_/Li silicates peaks in the Li 1s region and the Li_4_SiO_4_ peak in the Si 2p region remained unchanged, indicating their very high resistivity. At this stage, highly resistive species, such as newly formed Li_4_SiO_4_ and Li_2_O and initially present Li_2_CO_3_, as well as Li_1.20_Si, remained on the anode. The amount of Li consumed in the formation of Li_4_SiO_4_ and Li_2_O corresponds to 8% of the initial charge capacity, which is equivalent to 203 mAh g_Si_^–1^. In addition, the amount of Li trapped in Li_1.20_Si corresponds to 1143 mAh g_Si_^–1^, making the total 1346 mAh g_Si_^–1^, which is slightly less than the irreversible capacity for the first lithiation/delithiation cycle of 1477 mAh g_Si_^–1^, as shown in Figure 2(a). The origin of the remaining irreversible capacity of 131 mAh g_Si_^–1^ may be due to a possible error of about 5–10% in the correlation between the Si 2p peak position corresponding to Li_*y*_Si and Li content *y*, or to the presence of O-rich SiO_*x*_ at vicinity of the SiO_*x*_/LLZT interface, which leads to the formation of a larger amount of Li_4_SiO_4_ and Li_2_O.

In the second cycle, the Li_*y*_Si peaks in the Li 1s and Si 2p regions quasi-reversibly responded to the electrochemical lithiation/delithiation with the remaining spectral features related to Li_2_O, Li_2_CO_3_, and Li_4_SiO_4_ almost unchanged.

To elucidate the electrochemical lithiation/delithiation mechanism of the *a*-SiO_*x*_ thin-film electrode, the spectral changes during the first and second cycles were monitored in a stepwise manner. Figure 4 shows the time course of photoelectron spectra during the first lithiation and delithiation. The peak positions of each chemical species in the Li 1s and Si 2p_3/2_ regions were plotted as a function of capacity density as shown in Figure 5. As described above, a peak corresponding to Li_2_CO_3_ was observed at around 55.9 eV in the Li 1s region in a pristine state. In the Si 2p region, Si^0^, Si^3+^ and Si^4+^ peaks were observed at around 98.7, 101.7, and 102.7 eV, respectively.

**Figure 4:**
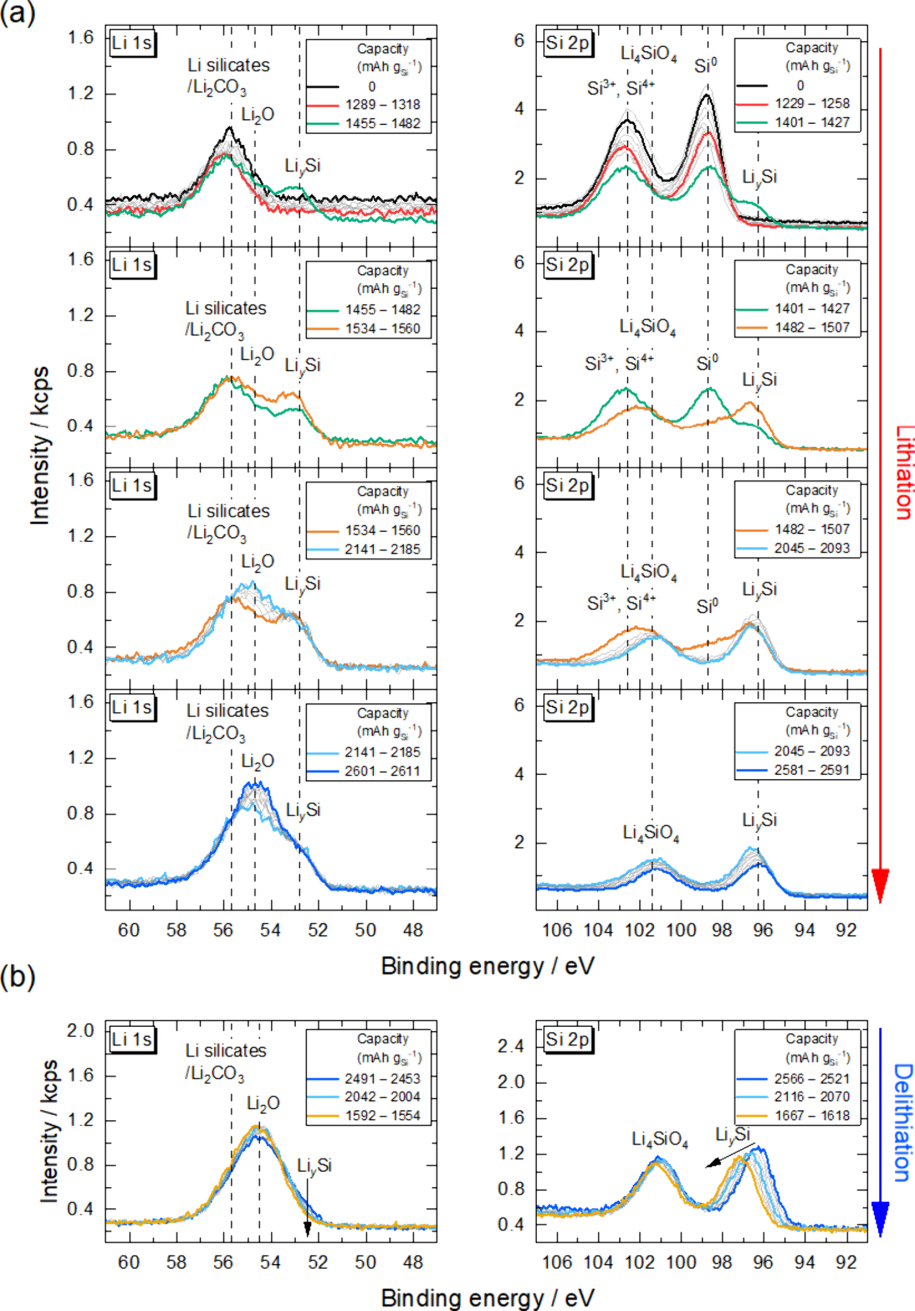
Li 1s and Si 2p photoelectron spectra of the *a*-SiO_*x*_ thin-film electrode in a Cu/SiO_*x*_/LLZT/Li ASS half-cell during the first (a) lithiation and (b) delithiation.

**Figure 5:**
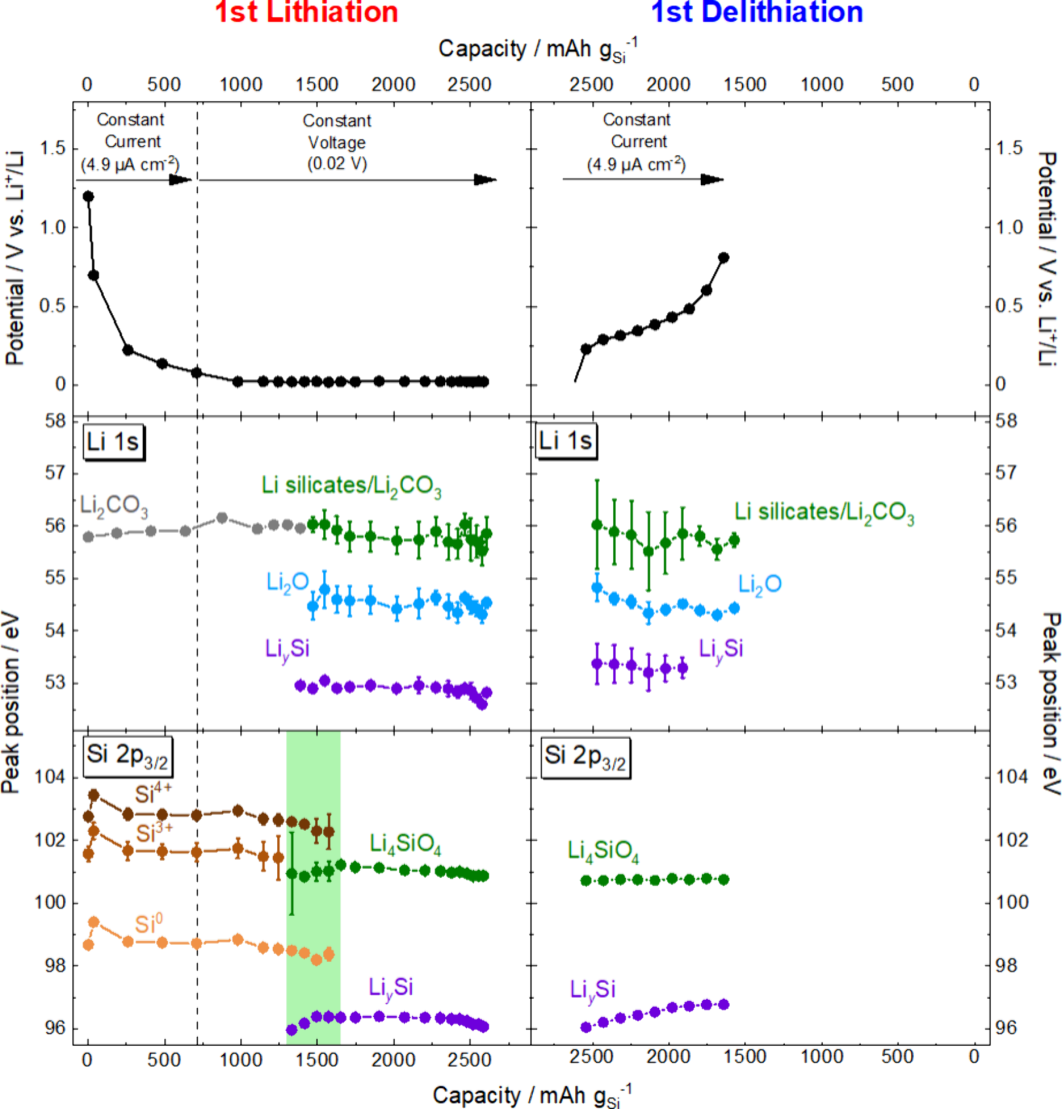
Potential profiles and Li 1s and Si 2p_3/2_ peak positions of each chemical species as a function of capacity density during the first lithiation and delithiation. The green area in Si 2p_3/2_ regions represent the region where Si^0^, Si^3+^, Si^4+^, Li_4_SiO_4_, and Li_*y*_Si coexist.

The peaks in Li 1s and Si 2p regions remained unchanged until the capacity density for the lithiation reached around 1318 mAh g_Si_^–1^ (red curves in Figure 4(a) and left panel of Figure 5). It is noted that the intensities of all of the peaks gradually decreased in this capacity range. This can be attributed to the fluctuation of incident photon flux because the shift of Si 2p_3/2_ peak attributed to the increase in Li content, was absent. Apart from Li_2_CO_3_ present from the beginning, Li_2_O and Li_4_SiO_4_, which are expected to attenuate the photoelectron signals from Si, have not yet been detected at this point.

When the capacity density reached around 1482 mAh g_Si_^–1^ (green curves in Figure 4(a) and left panel of Figure 5), new peaks corresponding to Li_*y*_Si suddenly appeared at 53.0 eV in the Li 1s region and at 96.4 eV in the Si 2p region as well as a Li silicate peak at around 101 eV in the Si 2p region. In addition, Li_2_O was also observed at around 54.5 eV in the Li 1s region. Based on the position of Li_*y*_Si peak at 96.4 eV in the Si 2p region, the composition is identified as Li_1.97_Si using the correlation in Figure S2.^27^

In the capacity density from 1401 to 1560 mAh g_Si_^–1^ (between green and orange curves in Figure 4(a) and left panel of Figure 5), the intensities of the Li_*y*_Si, Li silicates, and Li_2_O increased with decreasing intensities of Si^0^, Si^3+^, and Si^4+^ species in the Si 2p and Li 1s regions while maintaining the position of the Li_*y*_Si peak. When the capacity density reached 2093 mAh g_Si_^–1^ (light blue curve in Figure 4(a) and left panel of Figure 5), the Si 2p peaks corresponding to the Si^0^, Si^3+^, and Si^4+^ species disappeared completely. Simultaneously, the Li_*y*_Si peak started to shift monotonically from 96.4 to 96.1 eV at the capacity density of 2591 mAh g_Si_^–1^ (blue curve in Figure 4(a) and left panel of Figure 5), showing the lithiation of Li_2.02_Si to Li_2.53_Si (magnified view in Figure S4) based on the correlation in Figure S2 ^27^ in the Supporting Information. In the Li 1s region, the intensity of the Li_2_O peak at around 54.7 eV gradually increased when the capacity density exceeded 2185 mAh g_Si_^–1^ (between light blue and blue curves in Figure 4(a)), probably due to the side reactions of the topmost Li_*y*_Si with residual gases present in the vacuum chamber. In fact, the intensities of Li_*y*_Si and Li_4_SiO_4_ slightly decreased in the Si 2p region.

In our previous study on a ∼100 nm thick *a*-Si thin-film electrode, upon lithiation, the position of Si 2p peak drastically shifted from 99.1 to 98.3 eV due to the lithiation of *a*-Si to form slightly lithiated Li_*y*_Si. Even in the first Li 1s spectrum obtained 24 min after initiating the electrochemical lithiation (∼17 mAh g_Si_^–1^), Li species were already detected at the *a*-Si surface which is opposite to the Si/LLZT interface. This shows that the lithiation reaction occurred uniformly across the entire *a*-Si thin-film electrode. Then, the Li_*y*_Si peak monotonically shifted to a lower binding energy in response to the electrochemical charge, i.e., Li content *y* in Li_*y*_Si, until it reached 95.4 eV to form Li_3.5_Si.^27^ In our present study on the *a*-SiO_*x*_ thin-film electrode, however, the Si 2p_3/2_ peak remained unchanged until ∼1300 mAh g_Si_^–1^ and then Li_∼2,0_Si suddenly appeared at ∼1400 mAh g_Si_^–1^. This clear contrast suggests that the lithiation proceeded in a layer-by-layer manner due to the relatively low conductivity of SiO_*x*_; in the initial stage, the lithiation of SiO_*x*_ occurred at the vicinity of SiO_*x*_/LLZT interface which was located beyond the probing depth of XPS, and then gradually propagated into the bulk and approached the probing depth of XPS as the composition reached Li_∼2.0_Si to elongate an ion conductive pathway.

The Li_2.53_Si peak started to shift immediately after starting the subsequent delithiation (Figure 4(b) and the right panel of Figure 5). Thereafter, the Li_*y*_Si peak in the Si 2p region monotonically shifted to a higher binding energy up to 97.0 eV equivalent to Li_1.20_Si based on the correlation in Figure S3, and the intensity of the Li_*y*_Si peak at around 52.8 eV in the Li 1s region gradually decreased. Such a monotonic shift of Li_*y*_Si peak during delithiation was also observed in our previous study after shallow electrochemical lithiation of the *a*-Si thin-film electrode to form Li_2.3_Si.^27^ This result suggests that the delithiation reaction occurred uniformly across the *a*-SiO_*x*_ thin-film electrode once the ion conductive pathway composed of the Li_*y*_Si was formed in the first lithiation.

It is noteworthy that, in the *a*-Si thin-film electrode examined in our previous study, *a*-Li_3.45_Si was formed after lithiation up to around 3300 mAh g^–1^ at 0.02 V.^27^ In contrast, electrochemical lithiation of the *a*-SiO_*x*_ thin-film electrode spontaneously ceased at 2625 mAh g_Si_^–1^ at 0.02 V, likely due to the presence of highly resistive species. Such a self-limiting property may help prevent excessive lithiation and thereby improve cycle life, as deep lithiation leading to the formation of *c*-Li_3.75_Si causes rapid capacity fading due to the considerable volume change associated with phase transition to *a*-Li_*y*_Si during the subsequent delithiation.^27^

Figure S5 shows the time course of photoelectron spectra during the second lithiation and delithiation. The peak positions of each chemical species in the Li 1s and Si 2p_3/2_ regions were plotted as a function of capacity density as shown in Figure S6. The spectral features related to Li_*y*_Si quasi-reversibly responded to the electrochemical lithiation/delithiation; the intensity of Li_*y*_Si peak at 52.7 eV increased/decreased and the Si 2p_3/2_ peak of Li_*y*_Si shifted to a lower/higher binding energy during the lithiation/delithiation, confirming that the lithiation and delithiation uniformly occurred across the *a*-SiO_*x*_ thin-film electrode using the ion conductive Li_*y*_Si pathway.

The mechanism of the first electrochemical lithiation/delithiation of the *a*-SiO_*x*_ thin-film electrode on a LLZT sheet is shown in Figure 6 together with that of *a*-Si thin-film electrode in our previous study.^27^ In the pristine state (A 1), *a*-Si was distributed across the entire thin-film electrode. (A 2) Upon lithiation, *a*-Si was converted into a slightly lithiated *a*-Li_*y*_Si. (A 3, 4) Then, as the capacity density increased, the Li content *y* in Li_*y*_Si increased, and eventually Li_3.5_Si formed at the capacity of 3400 mAh g^–1^ where *a*-Li_*y*_Si and *c*-Li_3.75_Si coexisted, based on an assumption that all the electrical charge was consumed for the lithiation. In these processes (A 1–4), the composition of Li_*y*_Si was considered to be uniform throughout the thin-film electrode because of its relatively high ionic conductivity. (A 5) During the subsequent delithiation, Li ions were preferentially extracted from the *a*-Li_*y*_Si. (A 6) In the capacity range 1520–1920 mAh g^–1^ corresponding to the Li content *y* from 2.0 to 1.6, the phase transition from *c*-Li_3.75_Si to *a*-Li_*y*_Si occurred, as evident by the drastic shift of Li_*y*_Si peak as well as the positive d*Q*/d*V* peak around 0.41 V.^27^ (A 7) After the phase transition, the Li content *y* gradually decreased as evident by a monotonic shift of the Li_*y*_Si peak.^27^

**Figure 6:**
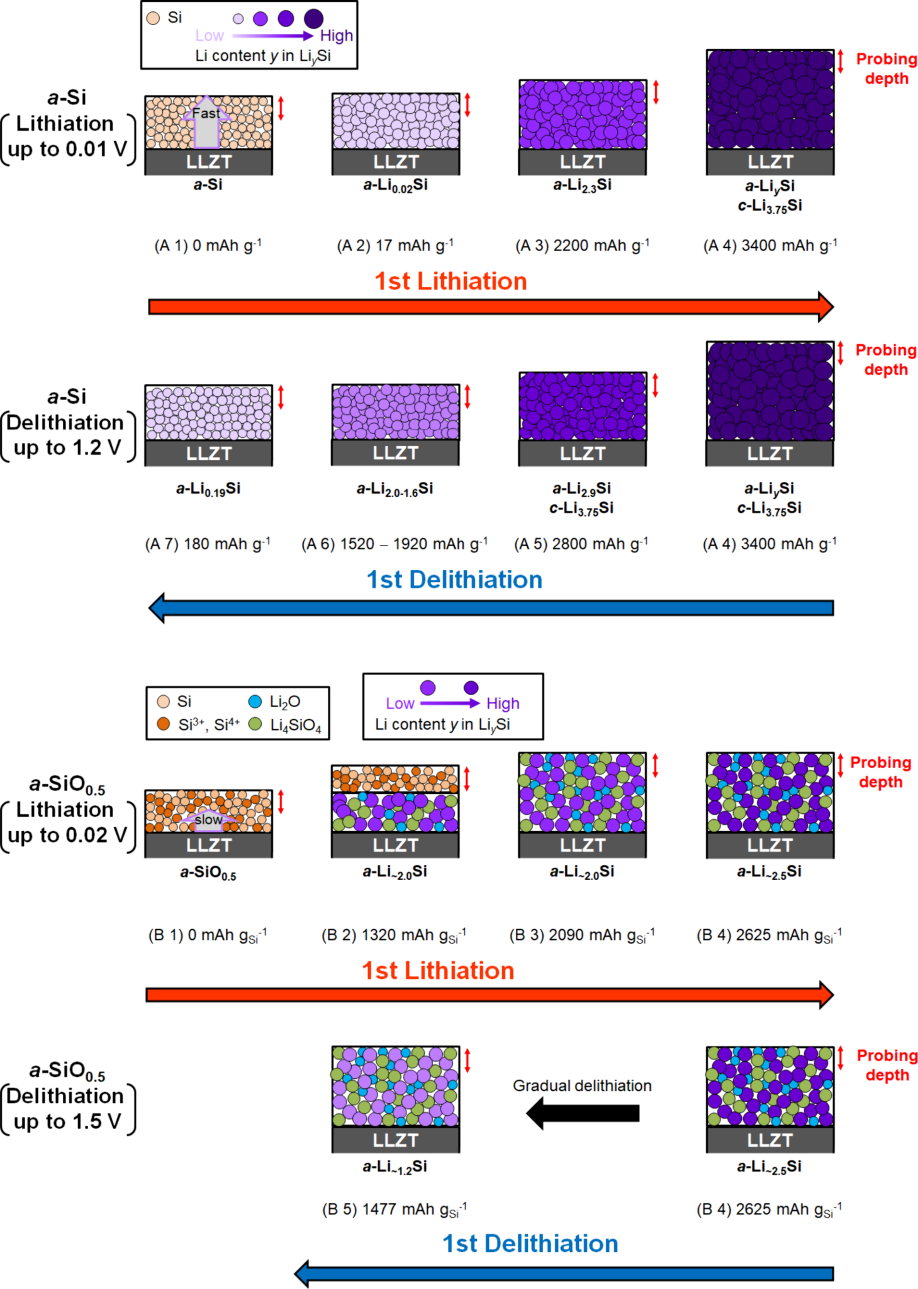
Electrochemical lithiation/delithiation mechanisms of (A) *a*-Si and (B) *a*-SiO_0.5_ thin-film electrodes deposited on the LLZT. (*a* = amorphous; *c* = crystalline).

(B 1) In the pristine state, the *a*-SiO_*x*_ thin-film electrode was mainly composed of Si^0^, Si^3+^, and Si^4+^ species. We assume that Si^0^ domains were uniformly distributed within the SiO_*x*_ thin film as previously reported.^14,21,42^ The oxygen content *x* in SiO_*x*_ was estimated to be ∼0.5 from the SEM-EDS and HAXPES spectra as shown in Figure S1 in the Supporting Information. (B 2) At the early stage, the lithiation occurred in the vicinity of the SiO_*x*_/LLZT interface which is beyond the probing depth of XPS, to form Li_*y*_Si, Li_2_O, and Li_4_SiO_4_, due to the intrinsically low ionic conductivity of Si oxide species.^22,43–45^ (B 3) The lithiation gradually propagated toward the surface opposite to the SiO_*x*_/LLZT interface, while forming an ion conductive pathway composed of Li_∼2.0_Si. (B 4) The lithiation reaction eventually reached the surface, which is the subject of XPS measurements, and further lithiation of the Li_∼2.0_Si occurred up to Li_∼2.5_Si until it reached the cut off voltage. (B 5) Delithiation proceeded uniformly across the thin film using the ion conductive Li_*y*_Si pathway.

In summary, electrochemical lithiation/delithiation processes of an *a*-SiO_*x*_ thin-film electrode deposited on a LLZT sheet were analyzed using *operando* XPS. Lithiation of SiO_*x*_ composed of Si^0^, Si^3+^, and Si^4+^ species first occurred in the vicinity of the SiO_*x*_/LLZT interface to form Li_*y*_Si, Li_2_O, and Li_4_SiO_4_, and then gradually propagated to the opposite surface which is the subject of XPS measurements, to elongate an ion conductive pathway composed of Li_∼2.0_Si. After the formation of the pathway, further lithiation into Li_∼2.0_Si occurred to form Li_∼2.5_Si. In the subsequent delithiation and cycles thereafter, lithiation/delithiation occurred while maintaining a spatially uniform composition across the *a*-SiO_*x*_ thin-film electrode via the pathway. Moreover, the Li content *y* in Li_*y*_Si reversibly responded to electrochemical lithiation/delithiation, as evident by the reversible shift of Si 2p peaks. In the case of *a*-Si thin-film electrode,^27^ electrochemical lithiation/delithiation occurred reversibly within the Li content range *y* = 0 to ∼2.3, leading to relatively high capacity retention. Once *c*-Li_3.75_Si was formed during deeper lithiation up to around 3400 mAh g^–1^, however, a phase transition from *c*-Li_3.75_Si to *a*-Li_*y*_Si occurred during the successive delithiation, resulting in rapid capacity fading due to significant volume changes.^27^ Unlike, electrochemical lithiation of SiO_*x*_ spontaneously ceased at a composition of Li_*y*_Si with lower Li content than that of pure *a*-Si, owing to the formation of highly resistive species such as Li_2_O, Li_2_CO_3_, and Li_4_SiO_4_. This self-limiting property is expected to help prevent excessive lithiation which may cause rapid capacity loss.

## Supplementary Material



## Notes

Detailed experimental procedures, oxygen content *x* in the *a*-SiO_*x*_ thin-film electrode in the pristine state estimated by XPS using an Al Kα source, HAXPES using a Cr Kα source, and SEM-EDS analysis, linear correlation between the position of Li_*y*_Si peaks in Si 2p region and the Li content *y* during the first lithiation and delithiation of the *a*-Si thin-film electrode, Li content *y* in Li_*y*_Si at the end of the first lithiation of the *a*-SiO_*x*_ thin-film electrode estimated from the linear correlation, and photoelectron spectra during the second lithiation and delithiation (PDF)
