# Supplementary material for: Electrochemical Lithiation and Delithiation of Amorphous Nonstoichiometric Silicon Oxide Thin-Film Electrode Studied by Operando X‑ray Photoelectron Spectroscopy
Source: J Phys Chem Lett. 2026 Feb 4;17(7):2181–90. doi: 10.1021/acs.jpclett.5c04065 (PMC12927016; doi:10.1021/acs.jpclett.5c04065)
Supplement: Supplementary file 1 [file jz5c04065_si_001.pdf]

Supporting Information of

"Electrochemical Lithiation and Delithiation of Amorphous Nonstoichiometric Silicon Oxide Thin-Film Electrode Studied by *Operando* X-ray Photoelectron Spectroscopy"

*Tsukasa Iwama[1,2], Ryosuke Sugimoto[3], Raimu Endo[1,2], Tsuyoshi Ohnishi[1], Masakazu Haruta[4], Takayuki Doi[3], and Takuya Masuda[1,2,]**

1. Research Center for Energy and Environmental Materials (GREEN), National Institute for Materials Science (NIMS), Tsukuba, Ibaraki 305-0044, Japan
2. Graduate School of Chemical Sciences and Engineering, Hokkaido University, Sapporo, Hokkaido 060-0810, Japan
3. Department of Molecular Chemistry and Biochemistry, Doshisha University, Kyotanabe, Kyoto 610-0321, Japan
4. Department of Electric and Electronic Engineering, Kindai University, Iizuka, Fukuoka 820-8555, Japan

***Corresponding Author:** Takuya Masuda MASUDA.Takuya@nims.go.jp

1. Experimental procedures.

An a-SiO_x thin film with a diameter of 10 mm and thickness of around 100 nm was deposited on a LLZT sheet (10 mm × 10 mm × 500 μm; Toshima Manufacturing Co., Ltd.) by radio frequency magnetron sputtering using Ar/O_2 gas mixtures.[1, 2] Then, a Cu layer serving as a current collector was deposited on the sputter-deposited SiO_x layer by direct current sputtering. During the Cu coating, the center area with dimensions of 10 mm × 4 mm was masked by a stainless-steel stencil plate to produce an uncoated SiO_x region for XPS measurements. Finally, a Li metal layer with a thickness of around 1.5 μm was formed on the other surface of the LLZT sheet by thermal evaporation to yield a Cu/SiO_x/LLZT/Li all-solid-state (ASS) half-cell, as shown in Figure 1.

The electrochemical lithiation/delithiation and XPS measurements were simultaneously carried out using an *operando* XPS system.[3-5] The ASS half-cell was mounted onto a sample holder in an Ar-filled glovebox and then transferred into the XPS apparatus (Kratos AXIS Nova, Shimadzu Corporation) without exposure to open air. The Cu layer on the a-SiO_x thin-film electrode and the Li metal layer were electrically connected to terminal A and B, respectively, with being insulated from each other by a polyimide film, as shown in Figure 1**Error! Reference source not found.**. After transferring the cell into the analysis chamber, terminal A and B were connected to a potentiostat (VSP-300, BioLogic Science instruments) at the outside by coaxial cables via a vacuum feedthrough, while terminal A was grounded with a hemispherical electron analyzer.

Electrochemical lithiation and delithiation of the ASS half-cell were carried out in constant-current (CC)–constant-voltage (CV) and constant-current (CC) mode, respectively. In the first lithiation with a CC–CV mode, the cell was first lithiated with a constant current of 4.9 μA cm^{-2} (~0.134 C, 1 C = 3579 mA g^{-1} for $Li_{3.75}Si$) until the cell voltage reached 0.02 V (109 min), and then the cell voltage was held constant at 0.02 V until the current density decreased to 0.49 μA cm^{-}

^{2} (546 min). In the delithiation with a CC mode, constant current of 2.45 μA cm^{-2} (~0.067 C) was applied until the cell voltage reached 1.5 V (285 min). The same procedure was applied in the subsequent lithiation/delithiation cycles, except that the current densities for CC and CV for lithiation were set to half of those for the first lithiation. In the present study, a low current density was used in order to obtain a sufficient number of photoelectron spectra during the lithiation/delithiation processes. In addition, it should be noted that, at higher current densities, the voltage immediately reaches the cutoff voltage due to a lower electric and ionic conductivity of SiO_x.

XPS measurements were performed in the analysis chamber kept under a pressure of 4×10^{-9} Torr. X-rays from a monochromatic Al Kα (1486.7 eV) source at a power of 300 W were incident to the exposed region of a-SiO_x thin-film electrode. The analysis area, takeoff angle, and pass energy of photoelectrons were fixed at 700×300 μm^2, 90°, and 80 eV, respectively. XPS measurements composed of Si 2p, C 1s, O 1s, and Li 1s regions were repeatedly applied to the a-SiO_x thin-film electrode throughout the lithiation and delithiation. The obtained spectra were calibrated by the hydrocarbon peak assignable to surface contamination at 285.0 eV in C 1s region as previously reported.[5] After the background subtraction by using the Shirley method, the spectra were fitted using the Voight function.[6] As for the Si 2p region, all the peaks were deconvoluted into Si $2p_{3/2}$ and $2p_{1/2}$ peaks and only the Si $2p_{3/2}$ peaks were used as the subject of discussion unless specified. In our previous study on an a-Si thin-film electrode sputter-deposited on a LLZT, a linear correlation between the peak position of Li_ySi in the Si 2p region and its Li content was obtained based on an assumption that all the electrochemical charge was consumed for the lithiation of Si.[5] In the present study, Li content in Li_ySi was determined based on the refined linear

correlation of our previous study on a-Si thin-film electrode[5] as shown in Table S2 and Figure S2 for the first lithiation, and Table S3 and Figure S3 for the first delithiation.

Hard X-ray photoelectron spectroscopy (HAXPES) measurements were performed in the analysis chamber equipped with a hemispherical analyzer (EW4000, Scienta Omicron, Inc.) kept under a pressure of 2×10^{-8} mbar.[7] Focused X-rays from a monochromatic Cr-Kα (5414.9 eV) source (ULVAC-PHI, Inc.) at a power of 50 W with a spot size of 200 μm were incident to the exposed area of a-SiO_x thin-film electrode. The takeoff angle, and pass energy of photoelectrons were fixed at 75° and 200 eV, respectively. HAXPES measurements composed of Si 2p and C 1s regions were applied to the pristine SiO_x thin-film electrode. The obtained spectra were calibrated and fitted in the same manner as those in the XPS measurements.

Elemental analysis of the a-SiO_x thin-film electrode in the pristine state was conducted using a SEM (SU8220, Hitachi) equipped with an energy dispersive X-ray spectroscopy (EDS, XFlash5060FQ, Bruker).

2. Estimation of oxygen content *x* in *a*-SiO*x*

The oxygen content x in the a-SiO_x thin film was estimated from SEM-EDS, XPS and HAXPES. According to elemental analysis using SEM-EDS, the oxygen content x in the SiO_x thin film in the pristine state was estimated to be 0.53.

Figure S1 (a) and (b) show the Si 2p photoelectron spectra of the SiO_x thin-film electrode in the pristine state obtained using Al Kα (XPS) and Cr Kα rays (HAXPES), respectively. The curve fitting analysis was performed using five symmetric Voigt functions[6] with each energy difference of 1 eV, corresponding to five species with the oxidation states of bulk Si (Si^0), Si_2O (Si^{1+}), SiO (Si^{2+}), Si_2O_3 (Si^{3+}), and SiO_2 (Si^{4+}).[8-11] The binding energy and the full width half maximum

(FWHM) of Si $2p_{3/2}$ peak due to Si^0 were set as variable parameters. In contrast, the FWHMs of peaks due to Si oxides such as Si^{1+}, Si^{2+}, Si^{3+}, and Si^{4+} species were fixed at the same value to reduce the number of refinement parameters. In addition, the intensity ratio and the difference of binding energy of the Si $2p_{1/2}$ to $2p_{3/2}$ peaks were fixed at 0.5 and 0.6 eV, respectively.[8-11] As a result, the best fit was obtained with Si^0, Si^{3+} and Si^{4+} components. The formation of silicon suboxides is commonly observed in nonstoichiometric silicon oxide and at the SiO_2/Si interface, and their chemical structures have been studied in the field of semiconductor technologies.[12-16]

The oxygen content x in SiO_x was estimated from the following equation (1);[8]

$$\text{Oxygen content } x = \frac{1.5 \times I_{\mathrm{Si}^{3+}} + 2 \times I_{\mathrm{Si}^{4+}}}{I_{\mathrm{Si}} + I_{\mathrm{Si}^{3+}} + I_{\mathrm{Si}^{4+}}} \qquad (1)$$

where I_{Si}, $I_{\mathrm{Si}^{3+}}$, and $I_{\mathrm{Si}^{4+}}$ are Si $2p_{3/2}$ peak intensities of Si^0, Si^{3+}, and Si^{4+}, respectively. The intensity ratio of Si^0, Si^{3+}, Si^{4+}, and estimated oxygen content x are summarized in Table S1. The estimated value of $x = 0.50$ from the photoelectron spectra using Cr Kα rays (HAXPES) was in good agreement with that of $x = 0.53$ estimated from SEM-EDS although value of $x = 0.86$ from the photoelectron spectra using Al Kα rays (XPS) was overestimated because Si surface is covered by a native oxide layer.

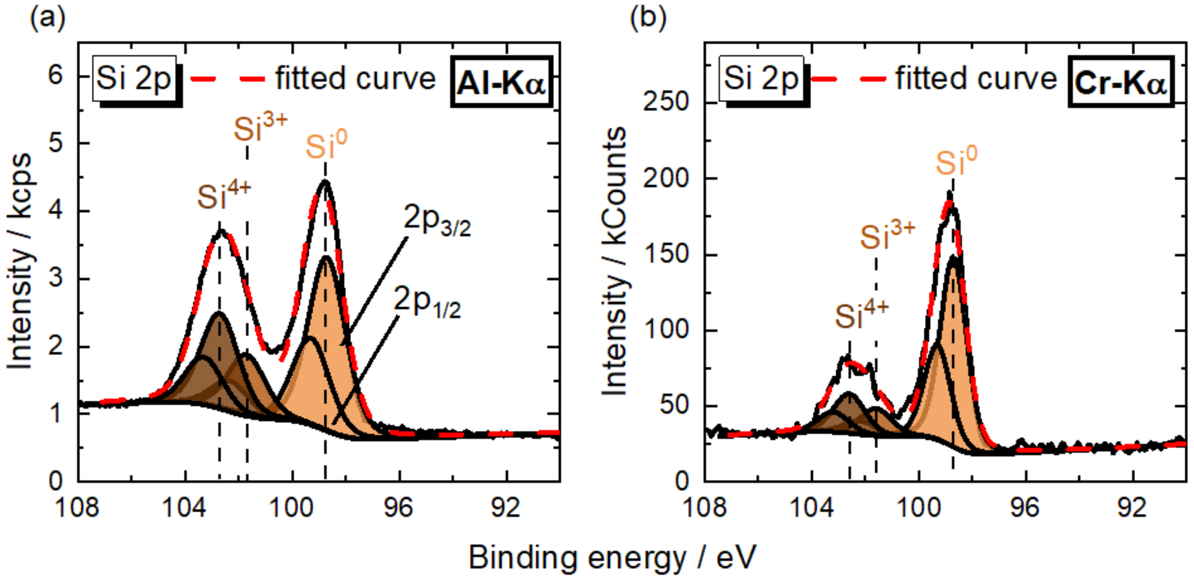

Figure S1. Si 2p photoelectron spectra of SiO_x thin film electrode in the pristine state obtained using (a) Al Kα and (b) Cr Kα rays.

Table S1. The oxygen content x in SiO_x estimated from the results of curve fitting of SiO_x thin-film electrode in the pristine state using equation (1).

Techniques \ Intensity ratio	I_{Si}	$I_{Si^{3+}}$	$I_{Si^{4+}}$	x in SiO_x
Al Kα (XPS)	0.52	0.19	0.29	0.87
Cr Kα (HAXPES)	0.72	0.12	0.16	0.50
EDS				0.53

3. The correlation between the position of Si 2p peaks due to Li_ySi and the Li content *y* during the first lithiation and delithiation of *a*-Si.[5]

In our previous study, electrochemical lithiation/delithiation reaction of an *a*-Si thin film electrode on a LLZT was dynamically analyzed by *operando* XPS.[5] The correlation between the position of Si 2p and deconvoluted Si $2p_{3/2}$ peaks due to Li_ySi and the Li content *y* obtained from electrochemical charge density during the first lithiation and delithiation are shown in Table S2 and S3, respectively. Figure S2 and S3 show the Si 2p and Si $2p_{3/2}$ peak positions as a function of Li content *y* in Li_ySi and linear approximations generated from those plots during the first lithiation and delithiation, respectively.

Table S2. Curve fitting results of Si 2p and Si $2p_{3/2}$ peaks due to Si^0/Li_ySi as a function of Li content y during the first lithiation.[5]

1st Lithiation	Si^0/Li_ySi			Si^0/Li_ySi	
y in Li_ySi	Si 2p	Si $2p_{3/2}$	y in Li_ySi	Si 2p	Si $2p_{3/2}$
0	99.07	98.95	1.77	96.68	96.52
0.018	98.27	98.27	1.88	96.61	96.45
0.128	97.71	97.58	1.99	96.55	96.38
0.237	97.68	97.53	2.10	96.49	96.30
0.347	97.57	97.42	2.20	96.40	96.23
0.456	97.48	97.33	2.31	96.35	96.19
0.566	97.39	97.21	2.42	96.25	96.11
0.675	97.33	97.15	2.53	96.25	96.06
0.785	97.26	97.09	2.64	96.15	95.97
0.894	97.24	97.04	2.75	96.13	95.94
1.00	97.16	97.01	2.86	96.02	95.87
1.11	97.06	96.92	2.97	95.98	95.83
1.22	97.02	96.83	3.08	95.93	95.79
1.33	96.90	96.75	3.19	95.95	95.78
1.44	96.85	96.71	3.30	95.87	95.72
1.55	96.79	96.63	3.41	95.74	95.59
1.66	96.77	96.58	3.50	95.36	95.22

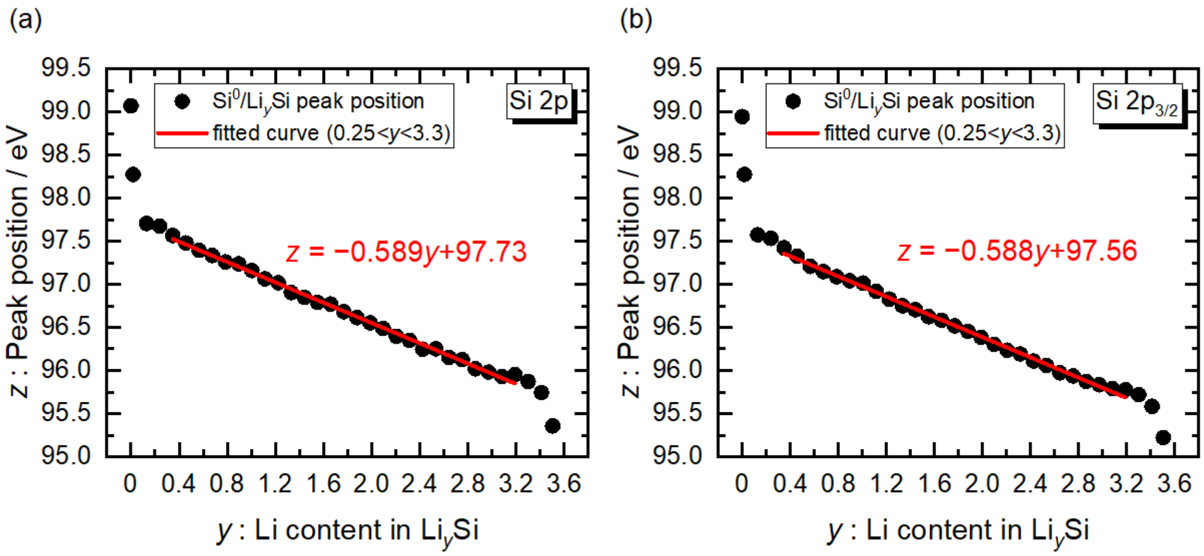

Figure S2. Positions of (a) Si 2p and (b) Si $2p_{3/2}$ peaks due to Si^0/Li_ySi as a function of Li content y during the first lithiation. Red lines are linear fits to the data in a range of $0.25 < y < 3.3$ [y : Li content in Li_ySi, z : Peak position / eV].

Table S3. Curve fitting results of Si 2p and Si2p$_{3/2}$ peaks due to Si^0/Li_ySi as a function of Li content y during the first delithiation.

1st Delithiation	Si^0/Li_ySi			Si^0/Li_ySi	
y in Li_ySi	Si 2p	Si 2p$_{3/2}$	y in Li_ySi	Si 2p	Si 2p$_{3/2}$
3.502	95.36	95.22	1.844	96.08	95.91
3.484	95.41	95.24	1.735	96.72	96.56
3.374	95.48	95.33	1.626	97.08	96.91
3.265	95.48	95.33	1.516	97.12	96.96
3.155	95.51	95.37	1.352	97.19	97.04
3.045	95.50	95.37	1.214	97.17	97.00
2.935	95.59	95.41	1.101	97.23	97.05
2.825	95.61	95.43	0.991	97.28	97.10
2.717	95.60	95.44	0.882	97.31	97.14
2.608	95.63	95.44	0.773	97.34	97.20
2.499	95.61	95.47	0.664	97.45	97.26
2.390	95.63	95.49	0.555	97.47	97.29
2.281	95.69	95.52	0.446	97.44	97.32
2.172	95.75	95.57	0.337	97.53	97.36
2.063	95.75	95.60	0.228	97.55	97.41
1.954	95.85	95.69	0.189	97.59	97.45

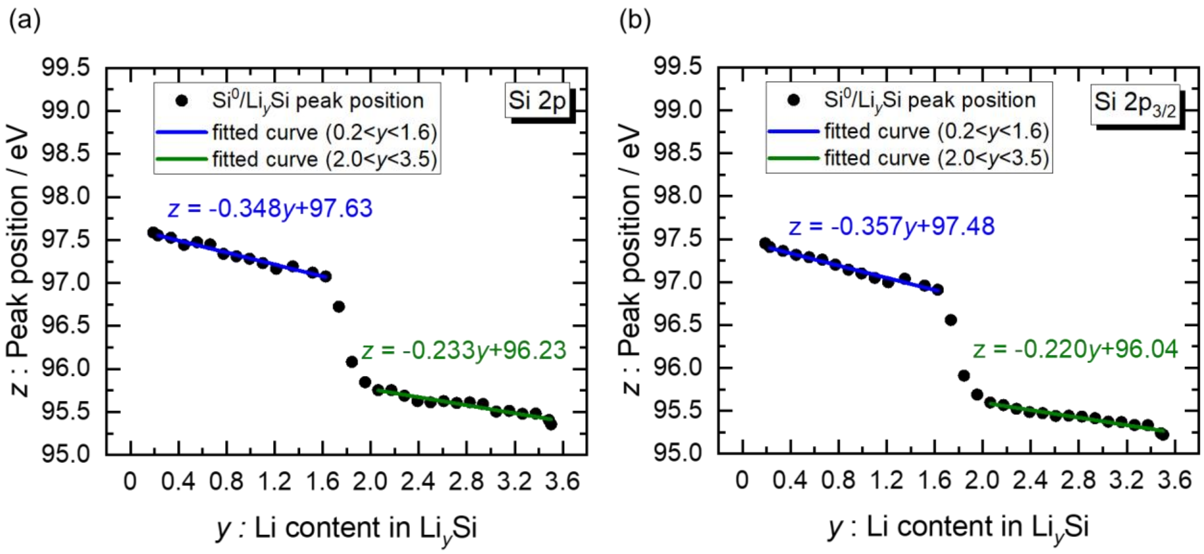

Figure S3. Positions of (a) Si 2p and (b) Si $2p_{3/2}$ peaks due to Si^0/Li_ySi as a function of Li content y during the first delithiation. Bule and green lines are linear fits to the data in ranges of $0.2 < y < 1.6$ and $2.0 < y < 3.5$, respectively [y : Li content in Li_ySi, z : Peak position / eV].

4. Si $2p_{3/2}$ peak positions corresponding to Li_ySi at the end of the first lithiation.

Figure S4 shows the Si $2p_{3/2}$ peak positions of each chemical species obtained by the curve fitting of XPS spectra of the a-SiO_x thin film (Figure 4) during the first lithiation, and Si $2p_{3/2}$ peak positions corresponding to Li_ySi at the end of the first lithiation. The Li_ySi peak gradually shifted from 96.4 eV at the capacity of 2093 mAh g_{Si}^{-1} to 96.1 eV at the capacity of 2591 mAh g_{Si}^{-1}.

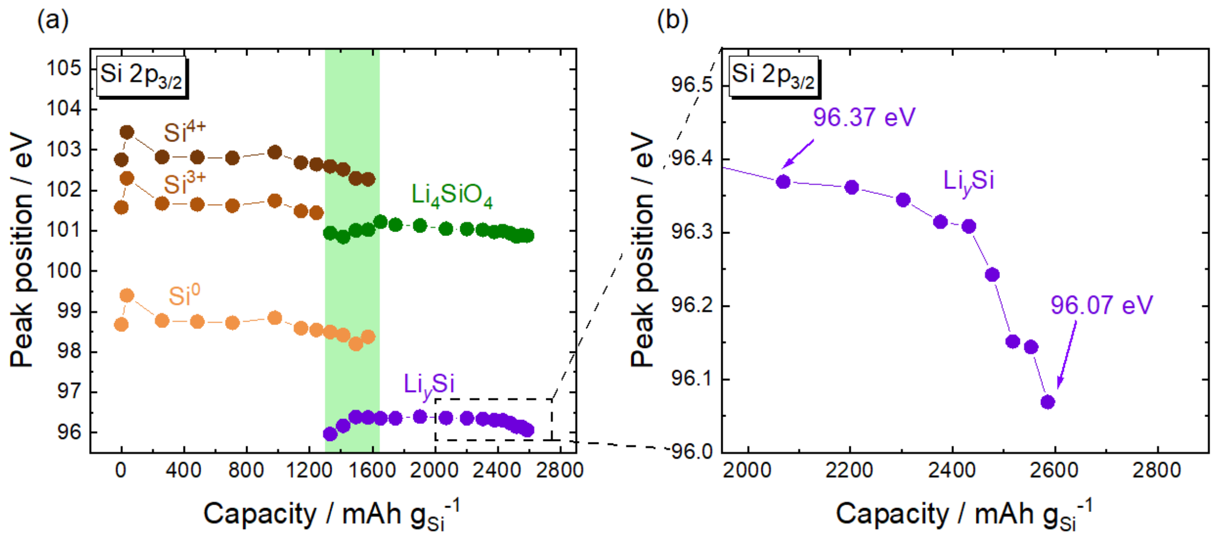

Figure S4. (a) Si $2p_{3/2}$ peak positions of each component with respect to capacity during the first lithiation. (b) Magnified graph of dashed box of (a). The green area shown in (a) and (b) represent the regions where Si^0, Si^{3+}, Si^{4+}, Li_4SiO_4, and Li_ySi coexisted.

In the first lithiation, the Li content y in Li_ySi was estimated by the following equation (2) (y: Li content y in Li_ySi, z: peak position / eV), as shown in Figure S2 (b);

$$z = -0.588y + 97.56 \ (0.25 < y < 3.3) \qquad (2)$$

Here, the Li_ySi peak position, z was 96.40 eV when the capacity density reached 1427 mAh g_{Si}^{-1} where Li_ySi and Li silicates were formed

$$\therefore y = 1.97$$

The Li_ySi peak position, z was 96.37 eV when the capacity density reached 2093 mAh g_{Si}^{-1} where almost all of the Si^0, Si^{3+}, and Si^{4+} were lithiated

$$\therefore y = 2.02$$

In addition, the Li_ySi peak position, z was 96.07 eV when the capacity density reached 2591 mAh g_{Si}^{-1}

$$\therefore y = 2.53$$

In the first delithiation, the Li content y in Li_ySi was estimated by the following equation (3), as shown in Figure S3 (b);

$$z = -0.357y + 97.48 \ (0.2 < y < 1.6) \tag{3}$$

Here, the Li_ySi peak position, z was 97.05 eV after the first delithiation

$$\therefore y = 1.20$$

5. Photoelectron spectra during the second lithiation and delithiation.

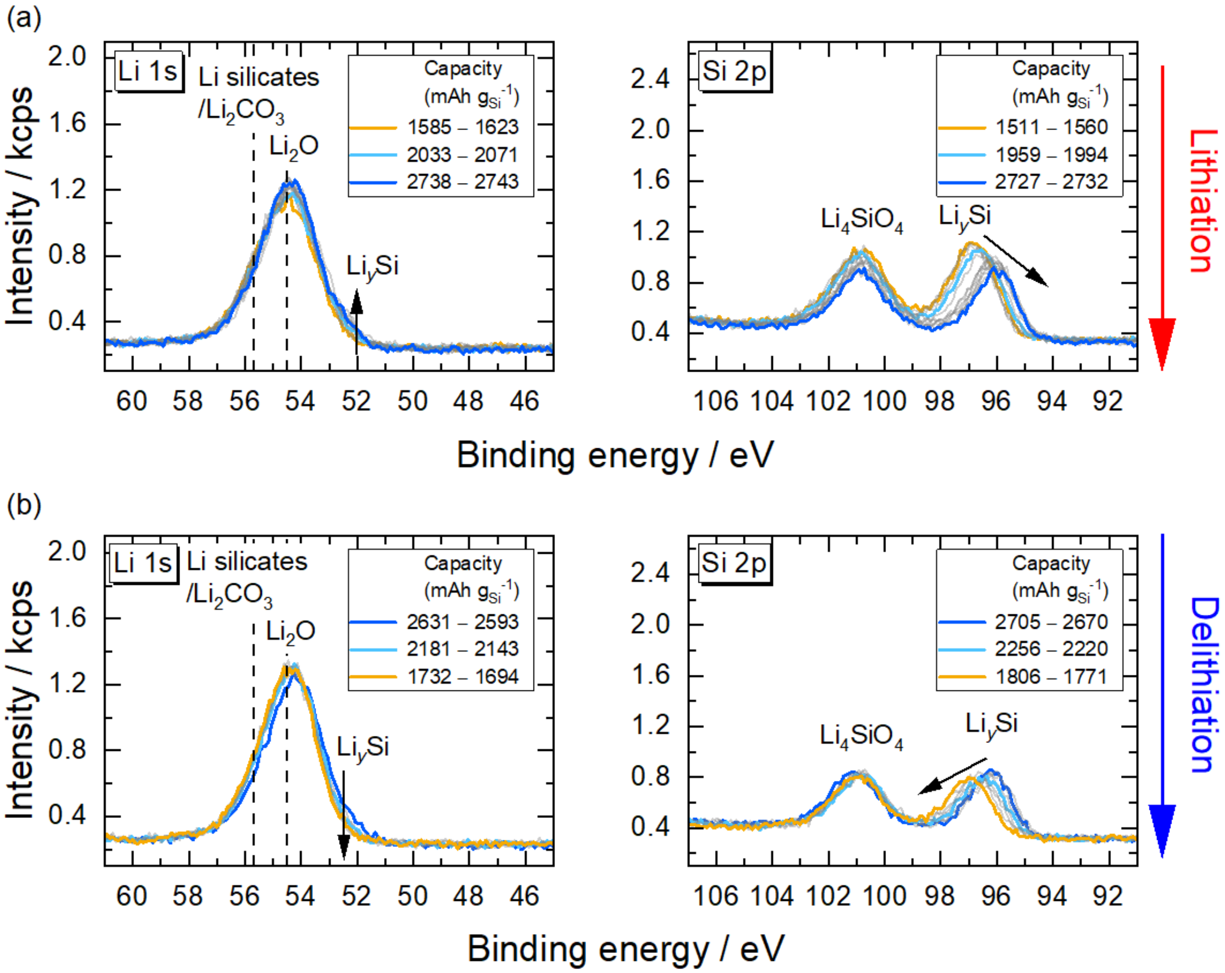

Figure S5. Li 1s and Si 2p photoelectron spectra of the *a*-SiO_x thin film electrode in a Cu/SiO_x/LLZT/Li ASS half-cell during the second (a) lithiation and (b) delithiation.

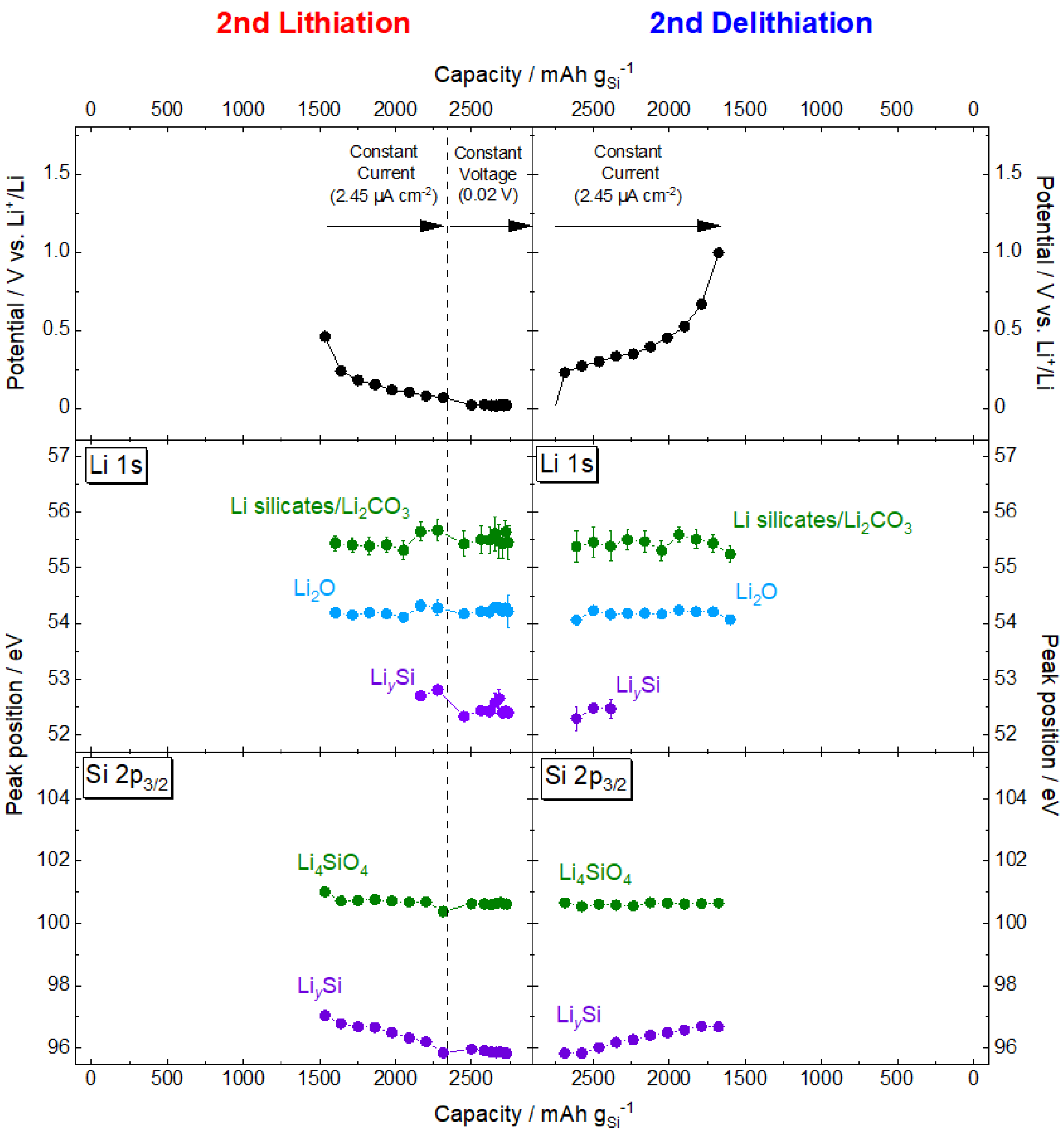

Figure S6. Potential profiles and Li 1s and Si 2p$_{3/2}$ peak positions of each chemical species as a function of capacity density during the second lithiation and delithiation.

References

(1) Sugimoto, R.; Marumoto, K.; Haruta, M.; Inaba, M.; Doi, T. Quantitative Evaluation and Improvement of Interfacial Li^+ Transfer Between SiO_x Electrode and Garnet‐Type Ta‐Doped $Li_7La_3Zr_2O_{12}$ Electrolyte. *ChemElectroChem* **2022**, *9* (17), e202200491. DOI: 10.1002/celc.202200491.

(2) Haruta, M.; Doi, T.; Inaba, M. Oxygen-Content Dependence of Cycle Performance and Morphology Changes in Amorphous-SiO_x Thin-Film Negative Electrodes for Lithium-Ion Batteries. *J. Electrochem. Soc.* **2019**, *166* (2), A258-A263. DOI: 10.1149/2.0861902jes.

(3) Endo, R.; Ohnishi, T.; Takada, K.; Masuda, T. Instrumentation for tracking electrochemical reactions by x-ray photoelectron spectroscopy under conventional vacuum conditions. *J. Phys. Commun.* **2021**, *5* (1), 015001. DOI: 10.1088/2399-6528/abd617.

(4) Endo, R.; Ohnishi, T.; Takada, K.; Masuda, T. In Situ Observation of Lithiation and Delithiation Reactions of a Silicon Thin Film Electrode for All-Solid-State Lithium-Ion Batteries by X-ray Photoelectron Spectroscopy. *J. Phys. Chem. Lett.* **2020**, *11* (16), 6649-6654. DOI: 10.1021/acs.jpclett.0c01906.

(5) Endo, R.; Ohnishi, T.; Takada, K.; Masuda, T. Electrochemical Lithiation and Delithiation in Amorphous Si Thin Film Electrodes Studied by Operando X-ray Photoelectron Spectroscopy. *J. Phys. Chem. Lett.* **2022**, *13* (31), 7363-7370. DOI: 10.1021/acs.jpclett.2c01312.

(6) Schmid, M.; Steinrück, H.-P.; Gottfried, J. M. A new asymmetric Pseudo-Voigt function for more efficient fitting of XPS lines. *Surf. Interface Anal.* **2014**, *46* (8), 505-511. DOI: 10.1002/sia.5521.

(7) Iwama, T.; Ohnishi, T.; Masuda, T. Operando Observation of Lithiation and Delithiation Reactions of a $LiCoO_2$-Li_3BO_3 Composite Electrode Formed on a $Li_{6.6}La_3Zr_{1.6}Ta_{0.4}O_{12}$ Solid

Electrolyte Sheet by Laboratory-based Hard X-ray Photoelectron Spectroscopy. *Electrochemistry* **2023**, *91* (11), 117005. DOI: 10.5796/electrochemistry.23-00090.

(8) Alfonsetti, R.; De Simone, G.; Lozzi, L.; Passacantando, M.; Picozzi, P.; Santucci, S. SiO_x surface stoichiometry by XPS: A comparison of various methods. *Surf. Interface Anal.* **2004**, *22* (1-12), 89-92. DOI: 10.1002/sia.740220122.

(9) Miyazaki, R.; Ohta, N.; Ohnishi, T.; Takada, K. Anode properties of silicon-rich amorphous silicon suboxide films in all-solid-state lithium batteries. *J. Power Sources* **2016**, *329*, 41-49. DOI: 10.1016/j.jpowsour.2016.08.070.

(10) Nguyen, C. C.; Choi, H.; Song, S.-W. Roles of Oxygen and Interfacial Stabilization in Enhancing the Cycling Ability of Silicon Oxide Anodes for Rechargeable Lithium Batteries. *J. Electrochem. Soc.* **2013**, *160* (6), A906-A914. DOI: 10.1149/2.118306jes.

(11) Bell, F. G.; Ley, L. Photoemission study of $SiO_x(0 \le x \le 2)$ alloys. *Phys. Rev. B* **1988**, *37* (14), 8383-8393. DOI: 10.1103/physrevb.37.8383.

(12) Keister, J. W.; Rowe, J. E.; Kolodziej, J. J.; Niimi, H.; Tao, H. S.; Madey, T. E.; Lucovsky, G. Structure of ultrathin SiO_2/Si(111) interfaces studied by photoelectron spectroscopy. *J. Vac. Sci. Technol., A* **1999**, *17* (4), 1250-1257. DOI: 10.1116/1.581805.

(13) Grunthaner, F. J.; Grunthaner, P. J.; Vasquez, R. P.; Lewis, B. F.; Maserjian, J.; Madhukar, A. Local atomic and electronic structure of oxide/GaAs and SiO_2/Si interfaces using high-resolution XPS. *J. Vac. Sci. Technol.* **1979**, *16* (5), 1443-1453. DOI: 10.1116/1.570218.

(14) Hollinger, G.; Himpsel, F. J. Probing the transition layer at the SiO_2-Si interface using core level photoemission. *Appl. Phys. Lett.* **1984**, *44* (1), 93-95. DOI: 10.1063/1.94565.

(15) Grunthaner, F. J.; Lewis, B. F.; Zamini, N.; Maserjian, J.; Madhukar, A. XPS STUDIES OF STRUCTURE-INDUCED RADIATION EFFECTS AT THE Si/SiO_2 INTERFACE. *IEEE Trans. Nucl. Sci.* **1980**, *27* (6), 1640-1646. DOI: 10.1109/TNS.1980.4331082.

(16) Barranco, A.; Yubero, F.; Holgado, J. P.; Caballero, A.; Gonzalez-Elipe, A. R.; Mejias, J. A. Structure and chemistry of SiO_x (x<2) systems. *Vacuum* **2002**, *67* (3-4), 491-499. DOI: 10.1016/S0042-207X(02)00218-X.
